# Plasma vascular endothelial but not fibroblast growth factor levels correlate with colorectal liver metastasis vascularity and volume

**DOI:** 10.1054/bjoc.1999.1033

**Published:** 2000-02-01

**Authors:** M M Davies, S K Jonas, S Kaur, T G Allen-Mersh

**Affiliations:** Department of Gastrointestinal Surgery, Imperial College School of Medicine, Chelsea & Westminster Hospital, 369 Fulham Road, London, SW10 9NH, UK

**Keywords:** colorectal cancer, VEGF, bFGF, angiogenesis, tumour volume

## Abstract

The extent to which plasma levels of angiogenic factors in healthy individuals and tumour volume-related variations in colorectal cancer affect the accuracy of circulating angiogenic factors as predictors of colorectal cancer vascularity is unknown. We used enzyme-linked immunosorbant assay to measure plasma vascular endothelial growth factor (VEGF) and basic fibroblast growth factor (bFGF) levels in colorectal liver metastasis (CLM) patients, and ‘no cancer’ controls. CLM volume was determined from computerized tomography scans, and tumour vessel count and vessel volume from anti-endothelial antibody-stained biopsies. There was a significant (*P* = 0.03) increase in plasma VEGF level in 29 CLM patients (median 180.3 pg ml^−1^, iqr 132.5–284.8 pg ml^−1^) compared with 19 controls (median 125.8 pg ml^−1^, iqr 58.2–235.9 pg ml^−1^). There were significant correlations between plasma VEGF and tumour vessel count (*r* = 0.66, *P* = 0.03), tumour vessel volume (*r* = 0.59, *P* = 0.03), and CLM volume (*r* = 0.53, *P* = 0.03). A VEGF level in the upper quartile of the plasma VEGF distribution had a 70% sensitivity and 75% specificity in predicting an upper quartile liver metastasis tumour vessel count. No relation was identified between CLM and plasma bFGF levels. Plasma VEGF level predicted CLM vascularity, despite an overlap with normal levels and tumour volume-related variations. © 2000 Cancer Research Campaign


*British Journal of Cancer* (2000) **82**(5), 1004–1008
© 2000 Cancer Research Campaign
DOI: 10.1054/ bjoc.1999.1033, available online at http://www.idealibrary.com on IDEAL®

### Plasma vascular endothelial but not fibroblast growth factor levels correlate with colorectal liver metastasis vascularity and volume

**MM Davies, SK Jonas, S Kaur and TG Allen-Mersh**

Department of Gastrointestinal Surgery, Imperial College School of Medicine, Chelsea & Westminster Hospital, 369 Fulham Road, London SW10 9NH, UK

**Summary** The extent to which plasma levels of angiogenic factors in healthy individuals and tumour volume-related variations in colorectal cancer affect the accuracy of circulating angiogenic factors as predictors of colorectal cancer vascularity is unknown. We used enzyme-linked immunosorbant assay to measure plasma vascular endothelial growth factor (VEGF) and basic fibroblast growth factor (bFGF) levels in colorectal liver metastasis (CLM) patients, and 'no cancer' controls. CLM volume was determined from computerized tomography scans, and tumour vessel count and vessel volume from anti-endothelial antibody-stained biopsies. There was a significant ($P = 0.03$) increase in plasma VEGF level in 29 CLM patients (median 180.3 pg $ml^{-1}$, iqr 132.5–284.8 pg $ml^{-1}$) compared with 19 controls (median 125.8 pg $ml^{-1}$, iqr 58.2–235.9 pg $ml^{-1}$). There were significant correlations between plasma VEGF and tumour vessel count ($r = 0.66$, $P = 0.03$), tumour vessel volume ($r = 0.59$, $P = 0.03$), and CLM volume ($r = 0.53$, $P = 0.03$). A VEGF level in the upper quartile of the plasma VEGF distribution had a 70% sensitivity and 75% specificity in predicting an upper quartile liver metastasis tumour vessel count. No relation was identified between CLM and plasma bFGF levels. Plasma VEGF level predicted CLM vascularity, despite an overlap with normal levels and tumour volume-related variations. © 2000 Cancer Research Campaign

**Keywords:** colorectal cancer; VEGF; bFGF; angiogenesis; tumour volume

Angiogenesis is critical for tumour growth (Folkman, 1990), and is controlled by a variety of angiogenic peptides and proteins – including vascular endothelial growth factor (VEGF) and basic fibroblast growth factor (bFGF). VEGF is a glycoprotein which can be produced by tumour cells (Leung et al, 1989; Dirix et al, 1997), and has a direct effect on the proliferation of endothelial cells within tumours (Senger et al, 1993). Increased tumour VEGF expression correlates with poor prognosis in colorectal (Kang et al, 1997), breast (Yamamoto et al, 1996), gastric (Maeda et al, 1996), ovarian (Yamamoto et al, 1997) and squamous (Eisma et al, 1997) carcinomas. The peptide bFGF also stimulates vascular endothelial cell proliferation, and bFGF expression has been identified in colorectal (Dirix et al, 1996), prostate (Meyer et al, 1995), cervical (Sluitz et al 1995*a*), breast (Sluitz et al 1995*b*), renal cell (Fujimoto et al, 1991), pancreatic and lung (Basilico and Moscatelli, 1992) cancers.

VEGF and bFGF can be detected in the circulation by enzyme-linked immunosorbant assay (ELISA) (Kondo et al, 1994), and measurement of their levels in the circulation might provide a non-invasive and repeatable means of obtaining information about tumour vascularity and response to anti-angiogenic therapies (Gasparini & Harris, 1995). Serum VEGF levels have been shown to correlate with stage of primary colorectal carcinoma (Kumar et al, 1998; Fujisaki et al, 1998), but it is not clear whether this is due to variations in bulk of disease or tumour angiogenicity. Similarly, increased serum bFGF levels are associated with primary colorectal carcinoma (Landriscina et al, 1998), but the extent to which these reflect tumour vascularity is unknown. In addition, the extent to which normal circulating VEGF and bFGF might reduce the ability of plasma levels to predict tumour vascularity has not been established.

The aim of the present study was to assess whether plasma levels of VEGF and bFGF could predict vascularity within colorectal liver metastases.

*Received 18 September 1998*
*Revised 21 September 1999*
*Accepted 20 October 1999*

*Correspondence to:* TG Allen-Mersh

### METHODS

### Patients studied, and blood and tumour sample processing

Ten millilitres peripheral venous blood were taken from patients with colorectal liver metastases in whom there was no evidence of extrahepatic disease on chest radiograph or abdominal computerized tomography (CT) scan, no history of chemotherapy treatment, and whose primary tumour had been removed more than 3 months previously.

The blood was collected into a potassium EDTA tube, centrifuged at 1500 rpm for 15 min and kept at –70°C until processing. Tumour vascularity was measured in a subgroup of these patients undergoing laparotomy for hepatic arterial cannulation (Allen-Mersh et al, 1994). A 5 ml volume liver metastasis biopsy was taken at the time of operation, rapidly frozen in isopentane and stored at –70°C for subsequent immunohistochemical staining.

Ten millilitres peripheral blood was taken prior to hernia repair from 'control' patients with no history of cancer and no current illnesses, and processed as above.

### Plasma VEGF measurement

Measurement was by solid phase ELISA (R&D Systems, UK) which detects both the secreted VEGF isoforms (121 and 165). One hundred microlitres of assay diluent was added to each well of a microtitre plate that had been pre-coated with an anti-VEGF monoclonal antibody. One hundred microlitres of the plasma sample was then pipetted into each well and incubated for 2 h at room temperature. VEGF present in the sample was bound by the immobilized antibody. The plate was then washed three times with wash buffer to remove unbound VEGF, 200 μl of enzyme-linked anti-VEGF polyclonal antibody added to the wells, and incubated for a second period of 2 h at room temperature to allow the secondary anti-VEGF antibody to bind to the immobilized VEGF. The plate was then washed again, 200 μl of antibody-linked enzyme substrate (50:50 hydrogen peroxide and tetramethylbenzidine) added, and incubated for 20 min at room temperature. The resulting yellow colour was proportionate to VEGF concentration. Following addition of 50 μl of stop reagent (2 M sulphuric acid) the 450-nm colour intensity within each well was measured using a spectrophotometer (Titertek Multiscan) to obtain a value for optical density. A VEGF standard dilution series was produced by serial dilution of a known quantity of VEGF (2000 pg $ml^{-1}$, 1000 pg $ml^{-1}$, 500 pg $ml^{-1}$, 250 pg $ml^{-1}$, 125 pg $ml^{-1}$, 62.5 pg $ml^{-1}$, 31.2 pg $ml^{-1}$). The optical density of these standard solutions was plotted against their concentrations to produce a standard curve which was then used to determine the VEGF concentration in pg $ml^{-1}$ within each plasma sample. The minimum detectable VEGF level was 9 pg $ml^{-1}$.

### Plasma bFGF assay bFGF was also measured using an ELISA technique (R&D Systems, UK) as above. A bFGF standard dilution series was produced by serial dilution of a known quantity of bFGF (640 pg $ml^{-1}$, 320 pg $ml^{-1}$, 160 pg $ml^{-1}$, 80 pg $ml^{-1}$, 40 pg $ml^{-1}$, 20 pg $ml^{-1}$, 10 pg $ml^{-1}$). Optical density of these standard solutions was plotted against their concentrations to produce a standard curve which was then used to determine the concentration of bFGF in each plasma sample assayed. The minimum detectable bFGF level was 7 pg $ml^{-1}$.

Duplicate aliquots from a single blood sample taken from each patient were assayed, and the mean concentration of the two samples was taken as the plasma level for that patient. The median intra-assay variation was 3.7% (iqr 1.3–8.0%) and the median inter-assay reproducibility (measured by repeat assay of eight samples) was 2.9% (0.8–12.0%).

### Liver metastasis volume measurement

Liver metastasis volume was measured as previously described (Dworkin et al, 1995). In brief, liver metastasis area was measured on each CT slice using a Konitron image analysis system (Imaging Associates, UK) and the volume for each slice then calculated by multiplying the area by the CT slice thickness. The volumes for all slices were then summed to obtain a total liver metastasis volume for each patient.

### Tumour vascularity assessment

Six-micrometre-thick tumour sections were cut by cryostat, transferred to polysine slides (75 × 25 × 1 mm; BDH, UK), fixed in acetone (BDH, UK) for 10 min and incubated for 5 min in 0.1% hydrogen peroxide in methanol (BDH, UK) to quench endogenous peroxide activity. Slides were then washed in Tris-buffered saline (TBS) (5 mM, pH 7.6) (Sigma, UK) for 5 min, and normal rabbit serum (Dako, UK) applied for 10 min, before incubation with 1:300 dilution primary anti-endothelial antibody (JC70, Dako, UK) for 30 min. Following incubation, the slides were rinsed in TBS and then dipped in 500-ml TBS containing 1 ml 1% BRIJ96 (10-ethyl ether) (Sigma, UK). The second antibody – a biotynylated rabbit anti-mouse IgG (Dako, UK) – was then applied for a further 30 min, followed by a further wash with TBS containing 1% BRIJ96 (as above). Streptavidin-conjugated peroxidase was applied for 30 min, followed by a third wash in TBS containing 1% BRIJ96. Slides were then transferred to Tris buffer, followed by incubation in DAB (diaminobenzidine) (Sigma, UK) solution for 5 min to stain the endothelial cells brown, washed in tap water and counterstained with Mayer’s haematoxylin (BDH, UK) for 1 min, dipped in 0.5% acid-alcohol (BDH, UK), blued in tap water and finally dipped in three concentrations (70, 80, 100%) of industrial methylated spirit (Hays, Leeds, UK) to dehydrate, clear and mount. This resulted in stained tissue sections in which blood vessels appeared brown and tumour nuclei blue. Positive controls were rat heart muscle which is abundant in vascular tissue, and negative controls were tumour sections stained without primary antibody.

Sections stained for vascularity with the anti-endothelial monoclonal antibody (JC70, Dako, UK) were examined at × 200 (× 10 eye-piece, × 20 objective) magnification using a Nikon Optiphot (Nikon, Japan) microscope. Random fields were obtained by defocusing the image, moving the slide and then refocusing. Two measures of vascularity were used: (1) Vessel count per $mm^2$ per microscope field which was calculated (Aherne and Dunnill, 1982) by summing the discrete brown-stained features seen within a 245 × 175 μm rectangular field set in the microscope eye-piece. The average of vessel counts per $mm^2$ in 40 randomly selected histological fields within each tumour section was used; (2) vessel volume (Chalkley, 1943) was derived by counting the number of dots falling on discrete brown-staining features, using a Chalkley grid (Graticules Ltd, UK) producing 25 dots set within the microscope eye-piece. Forty randomly selected fields were examined for each tumour section resulting in a total of 1000 points per tumour being assessed. Vessel volume was the percentage of dots overlying tumour vessels. The range of both vessel counts and vessel volumes in the 40 fields examined in each case was less than threefold greater than the median of all cases.

The study was approved by the Chelsea and Westminster Hospital Ethics Committee.

### RESULTS

### Patients

Forty-eight patients (19 ‘no cancer’ control and 29 colorectal liver metastasis patients) were studied. In the colorectal liver metastasis group, median liver metastasis volume was 380 ml (iqr 204–671).

### Tumour vascularity

Liver metastasis biopsies from 12 of the colorectal liver metastasis patients were examined. Median tumour vessel count was 28.43

© 2000 Cancer Research Campaign 

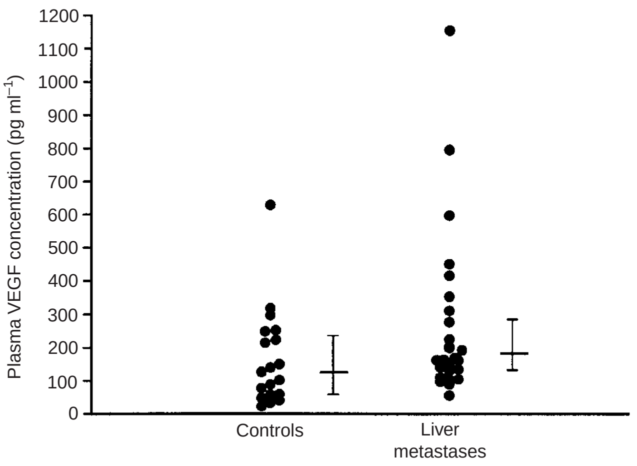

**Figure 1** The difference in plasma VEGF concentrations between patients with colorectal liver metastases and healthy 'no cancer' controls was relatively small (43% between medians). (Individual datapoints with medians and interquartile ranges.)

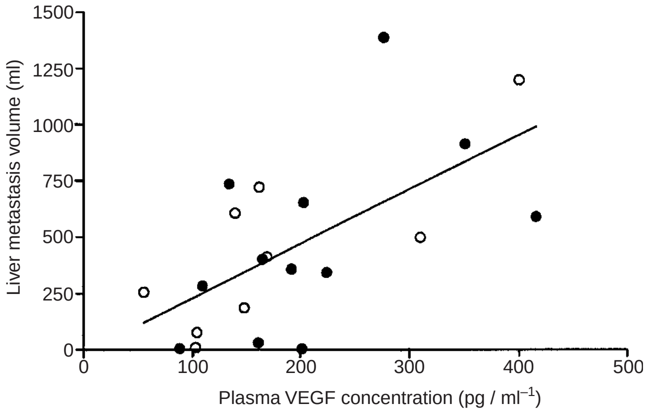

**Figure 2** There was a significant correlation ($r$ = 0.059, $P$ = 0.03) between plasma VEGF level and liver metastasis volume. (Solid symbols indicate patients in whom liver metastasis vascularity was also assessed.)

counts mm$^{-2}$ (iqr 20.33–35.71) and vessel volume 5.60% (4.55–10.20). Significant correlations (Spearman rank correlation test) between liver metastasis volume, and tumour vessel count ($r$ = 0.28, $P$ = 0.4) or vessel volume ($r$ = 0.16, $P$ = 0.65) were not detected.

### Plasma VEGF

Plasma VEGF levels (Figure 1) were significantly higher (Mann–Whitney $U$-test, $P$ = 0.03) in CLM patients (median 180.3 pg ml$^{-1}$, iqr 132.5–284.8) compared with controls (125.8, 58.2–235.9). There was a significant correlation (Spearman rank correlation test) between plasma VEGF and both tumour vessel count ($n$ = 12, $r$ = 0.66, $P$ = 0.03) and vessel volume ($n$ = 12, $r$ = 0.59, $P$ = 0.05). There was also a significant correlation ($n$ = 21, $r$ = 0.53, $P$ = 0.03) between plasma VEGF and liver metastasis volume (Figure 2).

### Plasma bFGF

No significant difference (Mann–Whitney $U$-test) between plasma bFGF level in CLM patients (median 95.2 pg ml$^{-1}$, iqr 44.5–191.7) compared with controls (112.8, 88.0–146.5) was demonstrated. Similarly, significant correlations (Spearman rank correlation tests) between plasma bFGF levels and liver metastasis volume ($n$ = 21, $r$ = 0.33, $P$ = ns), tumour vessel count ($n$ = 12, $r$ = 0.09, $P$ = ns) and vessel volume ($n$ = 12, $r$ = 0.42, $P$ = ns) were not demonstrated.

Significant correlations (Spearman rank correlation tests) were not detected between plasma VEGF and bFGF concentrations in either CLM ($n$ = 29, $r$ = 0.33, $P$ = ns) or 'no cancer' control ($n$ = 19, $r$ = 0.36, $P$ = ns) patients.

### DISCUSSION

Plasma VEGF in patients with colorectal liver metastases was significantly increased above the levels found in healthy controls, suggesting that VEGF associated with liver metastases was present in peripheral blood of these colorectal cancer patients. However, as reported previously (Yamamoto et al, 1996; Fujisaki et al, 1998; Kumar et al, 1998; Landriscina et al, 1998), we also detected VEGF in plasma from control patients. Although our colorectal liver metastasis patients had a substantial disease volume (median 380 ml), there was wide overlap between their plasma VEGF levels and those of control patients (Figure 1). Kumar et al (1998) have reported threefold greater levels in patients with metastatic colorectal cancer than those noted by us and by others (Fujisaki et al, 1998; Landriscina et al, 1998). Possible explanations for this difference are that Kumar et al reported serum VEGF levels which, unlike the plasma levels we have measured, may be influenced by VEGF release from platelets during blood clotting (Banks et al, 1998). In addition, unlike our liver metastasis patients, the patients with metastatic disease reported by Kumar et al also had unresected primary colorectal carcinomas that might also release VEGF. Our results suggested that normal plasma VEGF levels reduced the sensitivity of plasma VEGF as an indicator of colorectal liver metastases.

We did not find a significant increase in plasma bFGF level in the circulation of colorectal liver metastasis compared with control patients – unlike Landriscina et al (1998) who reported a doubling in mean serum bFGF level of primary colorectal cancer compared with control patients. However, in keeping with Landriscina et al's findings, we found no significant association between circulating bFGF and VEGF levels in either colorectal cancer or in control patients. This differs from Dirix et al (1997) who suggested in a report based on historical controls, that elevated serum VEGF and bFGF levels correlated in patients with a variety of metastatic cancers. Immunohistochemical studies of colorectal liver metastases have demonstrated only a 38.4% prevalence of positive staining for bFGF compared with 78.9% for VEGF (Terayama et al, 1996). One reason for the difference between our findings and those of Dirix et al (1996, 1997) could be that the pattern of angiogenic factors released into the circulation varies with tumour type.

The absence of a significant correlation between plasma bFGF and VEGF in 'no cancer' controls was in healthy persons without wounds and may not apply in non-cancer patients with healing wounds or conditions where angiogenesis is active.

 *© 2000 Cancer Research Campaign*

Immunohistochemical studies of colorectal cancer have suggested that the extent of tumour vascularity is related to local VEGF release (Takahashi et al, 1995). VEGF is a heparin-binding protein with higher levels within tissues than circulation, and plasma levels may not accurately reflect VEGF tissue activity. Our finding of a correlation between plasma VEGF level and colorectal liver metastasis vascularity has not been reported previously, but supports a previous report correlating serum VEGF level with breast cancer vascularity (Yamamoto et al, 1996). We also found a significant correlation between plasma VEGF level and colorectal liver metastasis volume. The absence of a significant positive correlation between liver metastasis volume and tumour vascularity suggested that the association between plasma VEGF level and colorectal liver metastasis volume was not explained by areas of high tumour vascularity being more likely to be biopsied in larger compared with smaller metastases. It was more likely that greater amounts of VEGF were released into the circulation in patients with larger colorectal liver metastases.

Comparisons of tumour vascularity with outcome have suggested that vessel density in areas of greatest neovascularization is an important predictor of poor survival (Weidner, 1998), although this is still controversial (Mayers et al, 1998). Thus it might be more clinically relevant for plasma VEGF level to be related to maximum rather than average tumour vascularity. However, it has been shown (Fox et al, 1995) that tumour vessel volume estimated by Chalkley's method (Chalkley, 1943) as used in the present study, also provides an estimate of breast cancer vascularity which correlates with survival. In addition, the finding of a less than threefold variability in vascularity between microscope fields within a biopsy suggested there was little vascular heterogeneity between the liver metastasis biopsy fields examined.

The present study examined a single 5 ml biopsy from one liver metastasis for vascularity, and these vascularity assessments may have been subject to sampling errors between liver metastases within the same patient, or between the vascular edge and avascular centre of the metastasis. Studies measuring total metastasis vascularity would be needed to assess whether tumour sampling variation was a source of error in estimating the relation between plasma VEGF and colorectal liver metastasis vascularity. Since not all colorectal cancers produce VEGF or bFGF (Terayama et al, 1996), the relationship between plasma levels and tumour vascularity might differ according to whether or not the angiogenic agent is produced by tumour cells. Further studies stratified by tumour VEGF or bFGF production would be required to evaluate this. The present correlations between plasma VEGF and colorectal liver metastasis vascularity, in patients who have not been selected by source of plasma VEGF and bFGF, are more relevant within a clinical context.

The findings of relatively substantial 'background' levels of plasma VEGF in healthy 'no-cancer' controls, together with variations in plasma levels by liver metastasis volume would be expected to reduce the accuracy of plasma VEGF as a predictor of vascularity in colorectal liver metastases. Despite these limitations, tumour vascularity correlated more strongly than liver metastasis volume with plasma VEGF, and a plasma VEGF level situated within the upper quartile of the plasma VEGF distribution was associated with a 70% sensitivity and 75% specificity in predicting a liver metastasis biopsy containing an upper quartile tumour vessel count.

These results provide support for studies assessing whether a plasma VEGF reduction could indicate tumour control with antiangiogenic treatments (Gasparini et al, 1996). We found no evidence of any relation between plasma bFGF level and colorectal liver metastases.

### ACKNOWLEDGEMENTS

MMD and SKJ were supported by Colon Cancer Concern

### REFERENCES

Aherne WA and Dunnill MS (1982) *Morphometry*, pp. 60–73. Edward Arnold: London

Allen-Mersh TG, Earlam S, Fordy C, Abrams K and Houghton J (1994) Quality of life and survival with continuous hepatic artery floxuridine infusion for colorectal liver metastases. *Lancet* **344**: 1255–1260

Banks RE, Forbes MA, Kinsey SE, Stanley A, Ingham E, Walters C and Selby PJ (1998) Release of the angiogenic cytokine vascular endothelial growth factor (VEGF) from platelets: significance for VEGF measurements and cancer biology. *Br J Cancer* **77**: 956–964

Basilico C and Moscatelli D (1992) The FGF family of growth factors and oncogenes. *Adv Cancer Res* **59**: 115–165

Chalkley HW (1943) Method for the quantitative morphological analysis of tissue. *J Natl Cancer Inst* **4**: 47–53

Dirix LY, Vermeulen PB, Hubens G, Benoy I, Martin M, DePooter C and Van Oosterom AT (1996) Serum basic fibroblast growth factor and vascular endothelial growth factor and tumour growth kinetics in advanced colorectal cancer. *Ann Oncol* **7**: 843–848

Dirix LY, Vermeulen PB, Pawinski A, Prove A, Benoy I, De Pooter C, Martin M and Van Oosterom AT (1997) Elevated levels of the angiogenic cytokines basic fibroblast growth factor and vascular endothelial growth factor in sera of cancer patients. *Br J Cancer* **76**: 238–243

Dworkin MJ, Burke D, Earlam S, Fordy C and Allen-Mersh TG (1995) Measurement of response to treatment in colorectal liver metastases. *Br J Cancer* **71**: 873–876

Eisma RJ, Spiro JD and Kreutzer DL (1997) Vascular endothelial growth factor expression in head and neck squamous cell carcinoma. *Am J Surg* **174**: 513–517

Folkman J (1990) What is the evidence that tumors are angiogenesis dependent? *J Natl Cancer Inst* **82**: 4–6

Fox SB, Leek RD, Weekes MP, Whitehouse RM, Gatter KC and Harris AL (1995) Quantitation and prognostic value of breast cancer angiogenesis: comparison of microvessel density, Chalkley count, and computer image analysis. *J Pathol* **177**: 275–283

Fujimoto K, Ichimori Y, Kakizoe T, Okajima E, Sakamoto H, Sugimura T and Terada M (1991) Increased serum levels of basic fibroblast growth factor in patients with renal cell carcinoma. *Biochem Biophys Res Commun* **180**: 386–392

Fujisaki K, Mitsuyama K, Toyonaga A, Matsuo K and Tanikawa K (1998) Circulating vascular endothelial growth factor in patients with colorectal cancer. *Am J Gastroenterol* **93**: 249–252

Gasparini G and Harris AL (1995) Clinical importance of the determination of tumor angiogenesis in breast carcinoma; much more than a new prognostic tool. *J Clin Oncol* **13**: 765–782

Kang S-M, Maeda K, Chung Y-S, Onoda N, Ogawa Y, Takatsuka S, Ogawa M, Sawada T, Kakata B, Nishiguchi Y, Ikehara T, Okuno M and Sowa M (1997) Vascular endothelial growth factor expression correlates with hematogenous metastasis and prognosis in colorectal carcinoma. *Oncol Rep* **4**: 381–384

Kondo S, Asano M, Matsuo K, Ohmori I and Suzuki H (1994) Vascular endothelial growth factor/vascular permeability factor is detectable in the sera of tumor-bearing mice and cancer patients. *Biochim Biophys Acta* **1221**: 211–214

Kumar H, Heer K, Lee PWR, Duthie GS, MacDonald AW, Greenman J, Kerin MJ and Monson JRT (1998) Preoperative serum vascular endothelial growth factor can predict stage in colorectal cancer. *Clin Cancer Res 4*: 1279–1285

Landriscina M, Cassano A, Ratto C, Longo R, Ippoliti M, Palazzotti B, Crucitti F and Barone C (1998) Quantitative analysis of basic fibroblast growth factor and vascular endothelial growth factor in human colorectal cancer. *Br J Cancer* **78**: 765–770

*© 2000 Cancer Research Campaign*

Leung DW, Cachianes G, Kuang W-J, Goeddel DV and Ferrara N (1989) Vascular endothelial growth factor is a secreted angiogenic mitogen. *Science* **246**: 1306–1309

Maeda K, Chung YS and Ogawa Y (1996) Prognostic value of vascular endothelial growth factor for gastric carcinoma. *Cancer* **77**: 858–863

Mayers MM, Seshadri R, Raymond W, McCaul K and Horsfall DJ (1998) Tumour microvascularity has no independent prognostic significance for breast cancer. *Pathology* **30**: 105–110

Meyer GE, Yu E, Siegal JA, Petteway JC, Blumenstein BA and Brawer MK (1995) Serum basic fibroblast growth factor in men with and without prostate carcinoma. *Cancer* **76**: 2304–2311

Senger DR, Van De Walter L, Brown LF, Nagy JA, Yeo KT, Yeo TK, Berse B, Jackman RW, Dvorak AM and Dvorak HF (1993) Vascular permeability factor (VPF, VEGF) in tumor biology. *Cancer Metastasis Rev* **12**: 303–324

Sluitz G, Tempfer C, Obermair A, Reinthaller A, Gitsch G and Kainz C (1995*a*) Serum evaluation of basic fibroblast growth factor in cervical cancer patients. *Cancer Lett* **94**: 227–231

Sluitz G, Tempfer C, Obermair A, Dadak C and Kainz C (1995*b*) Serum evaluation of basic fibroblast growth factor in breast cancer patients. *Anticancer Res* **15**: 2675–2678

Takahashi Y, Kitadai Y and Bucana CZ (1995) Expression of vascular endothelial growth factor and its receptor, KDR, correlates with vascularity, metastasis and proliferation of human colon cancer. *Cancer Res* **55**: 3964–3968

Terayama N, Terada T and Nakanuma Y (1996) An immunohistochemical study of tumour vessels in metastatic liver cancers and the surrounding liver tissue. *Histopathology* **29**: 37–43

Weidner N (1998) Tumoural vascularity as a prognostic factor in cancer patients: the evidence continues to grow [editorial comment]. *J Pathol* **184**: 119–122

Yamamoto Y, Toi M, Kondo S, Matsumoto T, Suzuki H, Kitamura M, Tsuruta K, Taniguchi T, Okamoto A, Mori T, Yoshida M, Ikeda T and Tominaga T (1996) Concentrations of vascular endothelial growth factor in the sera of normal controls and cancer patients. *Clin Cancer Res* **2**: 821–826

Yamamoto S, Koishi I, Kuroda H, Komatsu T, Nanbu K, Sakahara H and Mori T (1997) Expression of vascular endothelial growth factor (VEGF) in epithelial ovarian neoplasms: correlation with clinicopathology and patient survival and analysis of serum VEGF levels. *Br J Cancer* **76**: 1221–1227

*© 2000 Cancer Research Campaign*
